# The NF-κB pathway plays a vital role in rat salivary gland atrophy model

**DOI:** 10.1016/j.heliyon.2023.e14288

**Published:** 2023-03-08

**Authors:** Yang Yang, Yang Zi, Du Conglin, Zhang Chunmei, Hu Liang, Wang Songlin

**Affiliations:** a Department of Oral and Maxillofacial & Head and Neck Oncology, Capital Medical University School of Stomatology, Beijing Stomatological Hosptial, Captial Medical University, Beijing, 100050, China; b Salivary Gland Disease Center and Beijing Key Laboratory of Tooth Regeneration and Function Reconstruction, School of Stomatology and Beijing Laboratory of Oral Health, Capital Medical University, Beijing 100050, China; c Department of Oral and Maxillofacial Surgery, Capital Medical University School of Stomatology, Beijing, 100050, China; d Immunology Research Center for Oral and Systemic Health, Beijing Friendship Hospital, Capital Medical University, Beijing, 100069, China; e Department of Biochemistry and Molecular Biology, Capital Medical University School of Basic Medicine, Beijing, 100069, China; f Laboratory for Oral and General Health Integration and Translation, Beijing Tiantan Hospital, Capital Medical University, Beijing, 100069, China

**Keywords:** Salivary glands, Duct ligation, Histopathological change, RNA sequencing, TUNEL, terminal deoxynucleotidyl transferase dUTP nick end labeling, GO, Gene Ontology, KEGG, Kyoto Encyclopedia of Genes and Genomes, AB-PAS, Alcian blue–periodic acid Schiff staining

## Abstract

**Objective:**

The aim of this study was to explore the histopathological and genetic changes in the submandibular glands after duct ligation and provide important clues to functional regeneration.

**Design:**

We established a rat salivary gland duct ligation model and observed pathological changes in the rat submandibular gland on day 1 and weeks 1, 2, 3, and 4 using hematoxylin and eosin staining, Alcian blue–periodic acid Schiff staining, Masson staining, terminal deoxynucleotidyl transferase dUTP nick-end labeling (TUNEL), and immunohistochemical staining. RNA sequencing was performed on normal salivary glands and those from the ligation model after 1 week. Significantly differentially expressed genes were selected, and Gene Ontology (GO) and Kyoto Encyclopedia of Genes and Genomes (KEGG) pathway analyses were performed.

**Results:**

Apoptosis levels and histological and functional KEGG pathway analyses showed that injury to the salivary gland after ligation gradually increased. The TGF-β pathway was activated and promoted fibrosis. RNA sequencing results and further verification of samples at week 1 showed that the NF-κB pathway plays a vital role in salivary gland atrophy.

**Conclusions:**

Our results detailed the pathological changes in the submandibular gland after ligation and the important functions of the NF-κB pathway.

## 1 Introduction

Both the major and minor salivary glands produce and secrete digestive and protein-rich fluids in the oral cavity. However, three pairs of major salivary glands (parotid, submandibular, and sublingual) produce over 90% of the total saliva. Saliva assists in chewing and swallowing food, and digestive enzymes initiate digestion. Saliva is also important for speech and taste because it moisturizes the tongue and oral mucosa [1,2]. Healthy salivary glands are essential for maintaining oral and overall health. Salivary gland hypofunction is mainly manifested by a reduced salivary flow rate and reduced salivary secretion, which leads to oral mucosal dryness and discomfort, oral burning, and thirst. Long-term salivary gland hypofunction can alter the oral microenvironment, leading to severe dental erosion, mucosal lesions, or fungal infections [3]. Impairment of oral function may change a patient's eating preference, eventually leading to decreased masticatory muscle function, malabsorption, and nutritional deficiency [4].

Pathological conditions, such as obstructive factors, chronic heart failure, dementia, chronic kidney disease, psoriasis, and autoimmune diseases, such as Sjögren's syndrome and Hashimoto's disease, alter the function of salivary glands, which affects quality of life [[5], [6], [7], [8]]. Obstructive sialadenitis and sialolithiasis induce chronic or acute inflammation and swelling of the salivary gland due to obstruction of the salivary duct [9]. Radiation therapy is commonly used to treat head and neck cancer and can impair the salivary glands in the radiation therapy region, eventually leading to salivary gland dysfunction and atrophy [10,11]. Autoimmune diseases, such as Sjögren's syndrome, induce lymphocytic infiltration and fibrosis, leading to salivary gland hypofunction [12].

Current clinical treatments for salivary gland injury temporarily alleviate but do not restore the secretory function of the salivary gland [13]. Thus, experimental animal salivary gland injury models are required to develop new therapeutic strategies. After ligation of the main excretory duct, salivary glands in animal models appear to have symptoms similar to those of clinical patients. A gradual increase in intraductal pressure is the main cause of salivary gland atrophy with duct ligation, affecting the acinar and ductal epithelial cells [14]. Cell proliferation markers are upregulated when mesenchymal stem cells are used to treat the necrotic tissue of salivary glands in a rat submandibular gland duct ligation model [15]. The regeneration process is initiated by duct ligation as part of self-repair, thus activating Wnt/β-catenin signaling, which then promotes regeneration of the salivary gland [16].

Ligation of the salivary gland causes progressive atrophy of acinar cells. However, previous studies are mostly descriptive, and the pathological changes and mechanisms of the salivary glands have not been extensively reported. Damage to the parotid gland of a rabbit after ligation is divided into four stages with overlapping features [17]. In a mouse submandibular gland ligation model, fibrosis-induced TGF-β signaling was observed [18]. The level of transcription factor p63, which promotes regeneration, is upregulated after duct ligation and irradiation [19].

Above all, it is difficult to imagine any therapeutic approach for the treatment of the pathology presented; however, knowing the mechanism is the first step. In the present study, we aimed to investigate the histopathological and genetic changes caused by salivary gland injury in a rat submandibular gland ligation model to generate data to help develop better therapies for salivary gland injuries.

## 2 Materials and methods

### 2.1 Animal model

Male Wistar rats aged 10 weeks (200–250 g) were purchased from SPF Biotechnology Co. (Beijing, China) and kept in the animal house of Capital Medical University under standard conditions. The rats had free access to food and water, and ligation was performed after 1 week of habituation. Animal experiments were performed with the approval of the Animal Welfare Management Association (approval number: AEEI-2020-124).

The number of animals used in this project was the number necessary to obtain valid and meaningful results by referring to a relevant study [20]. Each group required at least six rats. Animals were randomly separated into six groups: the NT group (without animal treatment) and five experimental groups (1 day, 1 week, 2 week, 3 week, and 4 week, with ligation of the left duct of the submandibular gland). Ligation of the submandibular gland duct was performed by a skilled oral clinician. Animals were anesthetized with ketamine and the neck region was disinfected. The submandibular gland, duct, peripheral vasculature, and nerves were exposed. We then used a non-absorbable suture to ligate the main duct. Animals were observed and sacrificed at different timepoints. The salivary flow rate was tested by injecting pilocarpine intraperitoneally (5 mg/kg), and saliva was collected into sterile 1.5-mL tubes for 5 min [21]. The submandibular glands on both sides were collected and weighted, and then divided into several sections for further experiments.

### 2.2 Hematoxylin and eosin (HE) staining and alcian blue–periodic acid schiff staining (AB-PAS)

Fresh samples were collected and fixed in 4% formalin for 48 h. Then, after dehydration and wax embedding, samples were sliced into 5-μm sections. Sections were dehydrated using graded ethanol and vitrified with dimethylbenzene. Hematoxylin and eosin staining was performed to observe histological changes in the submandibular gland after ligation. The AB-PAS was performed to detect salivary gland function following the standard procedure (G1285, Solarbio, Beijing, China). We captured five different views in each section from different animals. The acinar cells and AB-PAS-positive areas were calculated using Image J Pro Plus (National Institutes of Health, Bethesda, USA) based on morphology.

### 2.3 Masson staining

The sections were dehydrated with gradient alcohol, as mentioned earlier. To check the degree of fibrosis, we used a Masson's trichrome staining kit (G1340, Solarbio). The nucleus was stained with hematoxylin, and muscle fiber was dyed with Masson red acid remix. Then, 2% glacial acetic acid, 1% phosphomolybdic acid aqueous solution, and aniline blue were used to stain collagenous fibers.

### 2.4 Terminal deoxynucleotidyl transferase dUTP nick-end labeling (TUNEL)

Paraffinized samples were sliced into 5-μm sections. TUNEL was performed using a One-Step TUNEL Apoptosis Assay Kit (C1090, Beyotime, Shanghai, China). Following the TUNEL reaction, the slides were treated with DAPI (F6057, Sigma, St. Loius, MO, USA) according to the manufacturer's instructions. Images were captured using a confocal microscope. The number of TUNEL-positive cells was calculated in six random fields per slide using ImageJ software.

### 2.5 Immunohistochemistry (IHC) and immunofluorescence (IF) staining

Paraffin sections for immunohistochemistry (IHC) after dewaxing were antigen-repaired using microwave heating. We then used 3% bovine serum albumin (BSA) to block non-specific antigens. Sections were incubated with primary antibodies (AQP5, ab78486, Abcam, Cambridge, UK; CD31, ab182981, Abcam; TGF-β1, ab92486, Abcam; IL-2, bs-4586R, Bioss, Beijing, China; IL-6, ab6672, Abcam; TNF-α, ab6671, Abcam; p-p65, bs-0982R, Bioss) overnight at 4 °C. The following day, different secondary antibodies were used for IHC (SP-9000, ZSGB-Bio, Beijing, China) and immunofluorescence (IF) (A32740, Thermo Fisher, Waltham, USA). We captured five different views in each section from 3 to 4 different animals. The positive areas were calculated using ImageJ Pro Plus.

### 2.6 Western blot detection

Submandibular glands were collected and protein was extracted within 2 h. The protein was preserved at −80 °C and quantified using the BCA assay (P1511-1, Applygen, Beijing, China). The primary antibodies used were p-IκKα (bs-3229R, Bioss), IκKα (A2062, ABclonal, Wuhan, China), p-IκBα (bs-18128R, Bioss), IκBα (10268-1-AP, Proteintech, Rosemont, USA), p-p65 (bs-0982R, Bioss), p65 (66535-1-Ig, Proteintech), and β-actin (AC026, ABclonal), which were incubated overnight at 4 °C after proteins were transferred to polyvinylidene fluoride membranes. Then, secondary antibodies (AS014 and AS003, ABclonal) were incubated the next day. We repeated our results three times and analyzed the data using GraphPad Prism 8.0 (GraphPad Software, San Diego, USA).

### 2.7 RNA sequencing

We randomly selected transcriptomes from normal submandibular gland samples (NT group, n = 3) and 1-week ligated samples (1w group, n = 3) for RNA sequencing. A comparison was performed to identify genes that were differentially regulated in the NT group and 1w group. Preparation of transcriptome libraries and sequencing were performed by OE Biotech Co., Ltd. (Shanghai, China). A P-value <0.05 and fold change >1.5 or <0.75 was set as the threshold for significantly differential expression. Gene Ontology (GO) and Kyoto Encyclopedia of Genes and Genomes (KEGG) pathway enrichment analyses were performed respectively using R based on hypergeometric distribution.

### 2.8 Statistical analysis

Results are presented as mean ± standard error of the mean (SEM). All graphs were generated using GraphPad Prism 9 software (GraphPad Software). One-way analysis of variance (ANOVA) was used to compare the differences among multiple groups, and the unpaired Student's t-test was used to compare two groups. Bonferroni's multiple comparisons test was used for post-hoc analysis of the ANOVA results. *P*-values <0.05 were considered statistically significant.

## 3 Results

### 3.1 Salivary gland function could partially recover at 1-week post-ligation

**Fig. 1:**
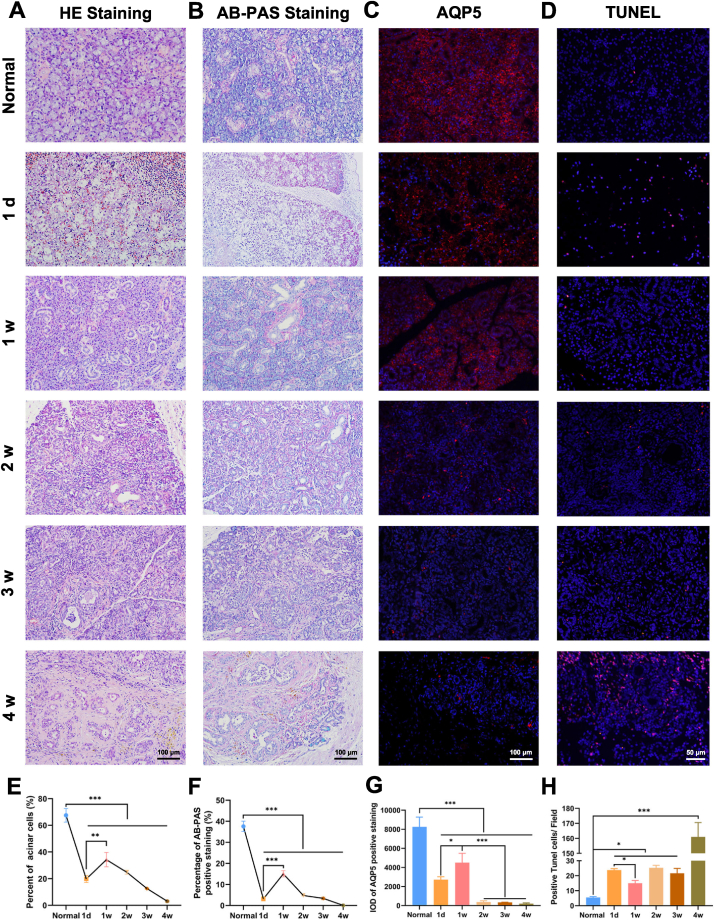
The histological changes of submandibular gland after duct ligation. H&E staining **(A)** and AB-PAS **(B)** staining of submandibular after ligation at NT, 1d, 1w, 2w, 3w, 4w. The expression of AQP5 **(C)** and TUNEL **(D)** were examined by immunofluorescent staining (red). Quantitative of acinar cells **(E)**, AB-PAS positive cells **(F)**, AQP5 positive cells **(G)**, positive TUNEL cells **(H)**. Data were shown as mean ± SEM, N = 6, (**E**, **F**) **p＜0.01 vs. Normal, (**G**, **H**) *p＜0.05, **p＜0.01, T-test and ANOVA. (For interpretation of the references to colour in this figure legend, the reader is referred to the Web version of this article.)

After duct ligation, the submandibular gland presented with characteristic injury. Because of the unilateral submandibular gland ligation, the weight of the ligated rats gradually increased. The salivary flow rate decreased significantly and did not recover. The mass of the submandibular gland increased significantly on day 1 and atrophied significantly on weeks 1, 2, 3, and 4 (Supplementary Fig. 1). The salivary gland acinar structures were obviously damaged at day 1, partially improved at week 1, and gradually worsened at weeks 2, 3, and 4. Interestingly, on day 1, the percentage of acinar cells was significantly reduced but partially recovered at week 1 (Fig. 1A, E). In addition, micrographs in a lower magnification were supplied to show the microscopic anatomy of submandibular gland (Supplementary Fig. 2). AB-PAS staining verified this phenomenon; samples had the smallest areas of blue staining on day 1 (Fig. 1B, F). IF for AQP5 was significantly lower on day 1 after ligation compared to the normal group. Compared with day 1, AQP5 expression increased at week 1 and then gradually decreased over the following weeks (Fig. 1C, G). Apoptotic levels were determined using TUNEL. The number of apoptotic cells in the submandibular gland at week 4 after ligation significantly increased (Fig. 1D, H).

### 3.2 Fibrosis in the submandibular gland was associated with the TGF-β pathway

**Fig. 2:**
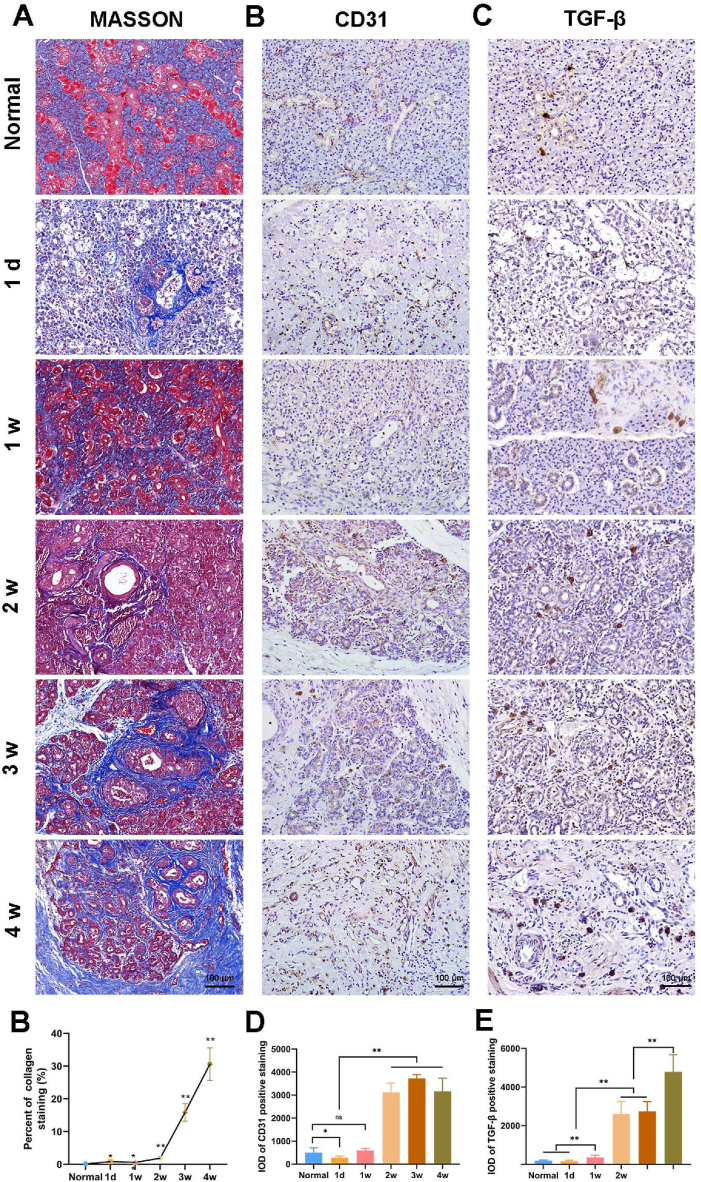
The fibro process of submandibular gland. The percent of collagen was examined by MASSON staining **(A)** and quantified **(B)**. The expression of CD31 **(C, D)** and TGF-β **(E, F)** were examined by immunohistochemistry staining and quantified. Data were shown as mean ± SEM, N = 6, (**B**) *p＜0.05, **p＜0.01 vs. Normal, (**D**, **F**) *p＜0.05, **p＜0.01, T-test and ANOVA.

The submandibular glands showed varying degrees of fibrosis after duct ligation. Masson staining showed that the collagen fibers (blue area) gradually increased and reached nearly 30% of the total area at week 4 (Fig. 2A and B). The microvasculature was checked using CD31 detection, which was present at a low level in the normal and ligation groups at day 1 and week 1. CD31 expression increased from week 2 after ligation and was maintained at a high level for the following weeks (Fig. 2C and D). The results of TGF-β IHC were similar to those of CD31, which indicated that fibrosis started at least 1 week after ligation (Fig. 2E and F).

### 3.3 Oxidative metabolism was inhibited and inflammation was enhanced 1-week post-ligation

**Fig. 3:**
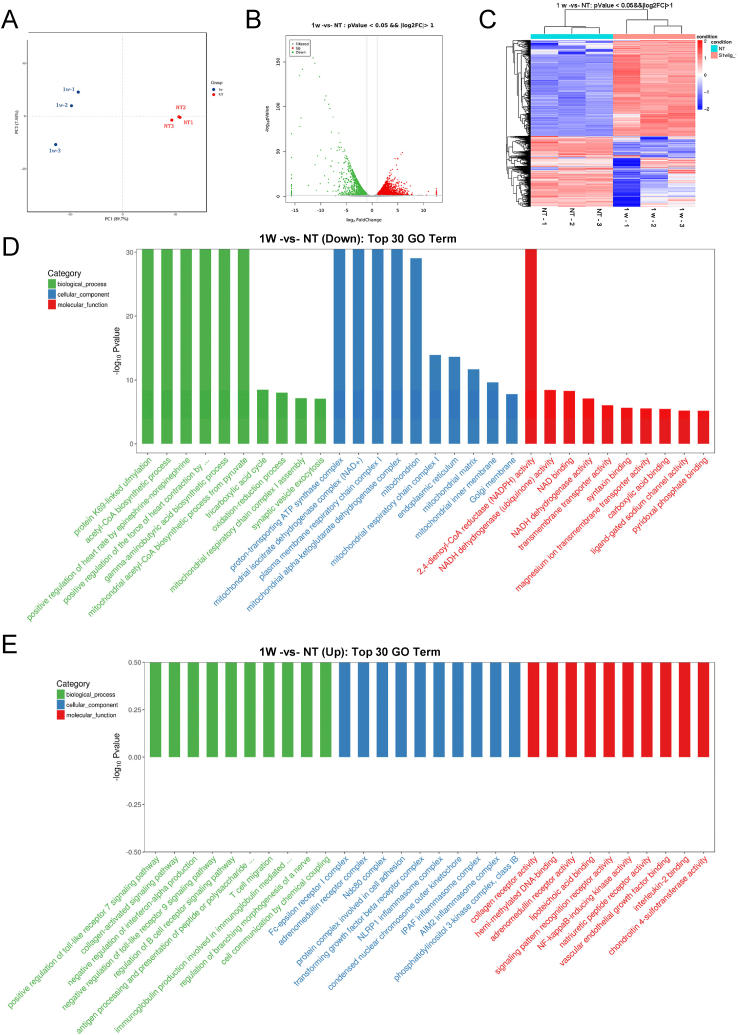
RNA sequencing of submandibular gland. **A:** normal group and 1 week group were separated by clustering analysis. **B:** volcano plot of differentially expressed mRNA transcripts between normal group and 1 week group. **C:** Heat map of hierarchical clustering. **D, E:** GO analysis about up regulate and down regulate gene between NT group and 1 week group.

In the present study, we observed pathological changes in the rat submandibular gland at different timepoints after ligation. Based on our previous results, we found that there was some recovery from gland injury after 1 week. Detecting gene expression at this timepoint could help to capture the mechanism of slight recovery. Thus, to explore the possible mechanisms that lead to pathological changes in the submandibular gland after duct ligation, 1w and NT submandibular gland samples were selected for RNA sequencing and further verification. Samples from these two groups were separated using clustering analysis (Fig. 3A). The PC values are provided in Supplementary Table 1. There were 2827 upregulated DEGs and 2074 downregulated DEGs between the 1w and NT groups. The different genes were screened out with a *P* value < 0.05 (Fig. 3B and C). There were 40 DEGs (including *klk1c10, LOC100911689, LOC103690048, klk1c12, LOC108348116,* and *LOC108348007*) that were only expressed in the NT group, and 84 DEGs (including *Ms4a4c, A2m, Ndnf, Sirpb2l1,* and *Lilrc2*) were only expressed in the 1w group. The top 30 upregulated and downregulated genes in the 1w group compared with the NT group are listed in Table 1, Table 2, respectively.

**Table 1:** Top 30 up-regulated genes ranked by P-value (Group 1w Vs. NT).

Rank	Gene ID	BaseMean_ NT	BaseMean_ 1 w	Fold Change	Product
1	Card14	24.37094	1457.178	59.79163	caspase recruitment domain family, member 14
2	Sfrp4	30.92965	1533.424	49.57781	secreted frizzled-related protein 4
3	Lox	70.8143	2198.942	31.05224	lysyl oxidase
4	Col3a1	6564.997	145760.8	22.20271	collagen type III alpha 1 chain
5	Adam8	56.24188	1814.877	32.26914	ADAM metallopeptidase domain 8
6	Slpi	40.04283	1078.521	26.93419	secretory leukocyte peptidase inhibitor
7	Plat	117.2446	1858.783	15.85388	plasminogen activator, tissue type
8	Col1a1	4751.944	144127.4	30.33019	collagen, type I, alpha 1
9	Ccdc80	445.7646	5637.83	12.64755	coiled-coil domain containing 80
10	Col14a1	391.1041	4374.337	11.18459	collagen type XIV alpha 1 chain
11	Pdpn	74.33305	1012.349	13.61909	podoplanin
12	Csf2rb	103.7653	1210.181	11.66267	colony stimulating factor 2 receptor beta common subunit
13	Adamts2	199.8993	2053.626	10.2733	ADAM metallopeptidase with thrombospondin type 1 motif, 2
14	Ikbke	120.0403	1315.223	10.95651	inhibitor of kappa light polypeptide gene enhancer in B-cells, kinase epsilon
15	Fbn1	1374.969	11162.87	8.118633	fibrillin 1
16	Vcan	86.56211	1957.633	22.61536	versican
17	C1s	950.3119	7485.324	7.876703	complement component 1, s subcomponent
18	Pxdn	532.552	5208.372	9.780026	peroxidasin homolog (Drosophila)
19	Gpc6	78.11378	831.0662	10.63917	glypican 6
20	Loxl1	243.3377	2635.562	10.83088	lysyl oxidase-like 1
21	Igfbp4	1199.774	9020.645	7.518617	insulin-like growth factor binding protein 4
22	C7	1171.485	8762.281	7.479636	complement component 7
23	Cpz	36.34474	780.234	21.46759	carboxypeptidase Z
24	C6	82.94242	824.5968	9.941798	complement component 6
25	Atp10d	157.9386	1348.714	8.539483	ATPase phospholipid transporting 10D (putative)
26	Mmp11	45.0332	889.7999	19.75875	matrix metallopeptidase 11
27	Sparc	4994.669	41830.69	8.375068	secreted protein acidic and cysteine rich
28	Rarres2	142.2405	1190.954	8.372821	retinoic acid receptor responder 2
29	Clec9a	6.49833	205.7704	31.66512	C-type lectin domain family 9, member A
30	Napsa	22.93218	343.8148	14.99268	napsin A aspartic peptidase

**Table 2:** Top 30 down-regulated genes ranked by P-value (Group 1w Vs. NT).

Rank	Gene ID	BaseMean_ NT	BaseMean_ 1 w	Fold Change	Product
1	Mucl1	85806.17	8.944232	0.000104	mucin-like 1
2	Klk1c2	90722.82	1.749959	1.93E-05	kallikrein 1-related peptidase C2
3	LOC102555445	1,402,943	484.0566	0.000345	16.5 kDa submandibular gland glycoprotein-like
4	Grpcb	2,407,620	1063.077	0.000442	glutamine/glutamic acid-rich protein A
5	Spt1	1,366,636	115.97	8.49E-05	salivary protein 1
6	Klk1c8	7051.769	0.38888	5.51E-05	kallikrein 1-related peptidase C8
7	Prol1	1,410,088	1158.573	0.000822	proline rich, lacrimal 1
8	RGD1306750	478941.2	948.305	0.00198	LOC362451
9	Egf	10135.68	17.20373	0.001697	epidermal growth factor
10	Klk1b3	638422.4	1847.534	0.002894	kallikrein 1-related peptidase B3
11	Car6	548052.7	1849.316	0.003374	carbonic anhydrase 6
12	Pip	1,004,664	5215.742	0.005192	prolactin induced protein
13	Klk1c9	57634.75	4.083237	7.08E-05	kallikrein 1-related peptidase C9
14	Klk1	6496.138	0.385784	5.94E-05	kallikrein 1
15	Mup5	8251.522	33.49003	0.004059	major urinary protein 5
16	Bpifa2f	256542.4	2885	0.011246	BPI fold containing family A, member 2F
17	Klk1c10	670.9832	0	0	kallikrein 1-related peptidase C10
18	Azgp1	3264.113	44.24335	0.013554	alpha-2-glycoprotein 1, zinc-binding
19	Slc5a11	1121.351	11.54561	0.010296	solute carrier family 5 member 11
20	Slc12a8	2103.824	32.77733	0.01558	solute carrier family 12, member 8
21	Unc5a	1304.028	15.83874	0.012146	unc-5 netrin receptor A
22	LOC259244	10902.86	42.71171	0.003917	alpha-2u globulin PGCL3
23	Bhlha15	10606.22	249.9687	0.023568	basic helix-loop-helix family, member a15
24	Cmah	6552.017	83.09775	0.012683	cytidine monophospho-N-acetylneuraminic acid hydroxylase
25	Aqp5	10217.58	242.361	0.02372	aquaporin 5
26	Gbgt1	403.1218	0.38888	0.000965	globoside alpha-1,3-N-acetylgalactosaminyltransferase 1
27	Snhg11	741.4099	6.682069	0.009013	small nucleolar RNA host gene 11
28	Car2	5899.083	150.8749	0.025576	carbonic anhydrase 2
29	Rasd2	1920.186	37.56411	0.019563	RASD family, member 2
30	Tesc	3442.492	100.8041	0.029282	tescalcin

**Fig. 4:**
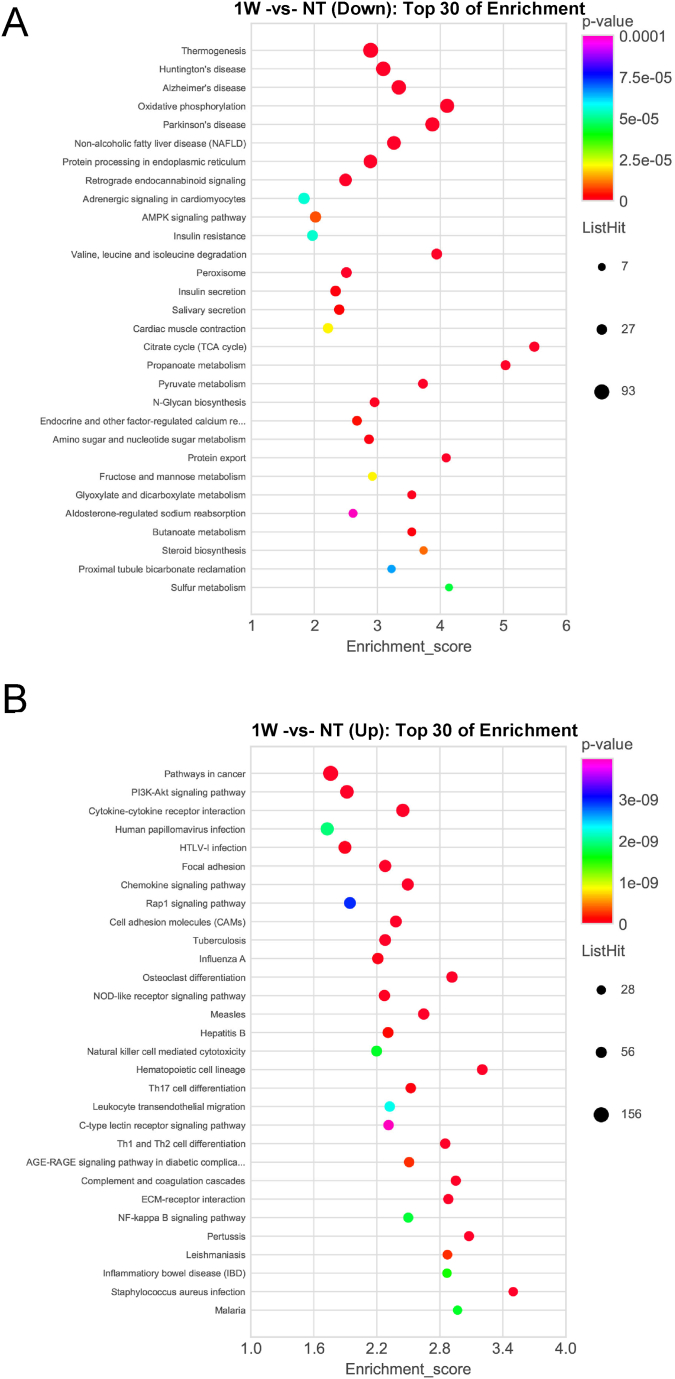
KEGG analysis between NT group and 1 week group. **A, B:** KEGG analysis about up regulate and down regulate gene showed that salivary secretion function between 1 week group normal group.

The downregulated genes included those encoding major salivary families of specific secretory proteins, like *Spt1* and *Csap1*. The expression of tissue-specific genes, such as kallikrein-related genes (*Klk1c2, Klk1c8, Klk1b3, Klk1c9, Klk1, Klk1c6, Klk15,* LOC687880, *Klk1c3*) decreased in the 1w group (Table 1). These genes mainly participate in processes such as protein K69-linked methylation, acetyl-CoA biosynthesis, the proton-transporting ATP synthase complex, the mitochondrial isocitrate dehydrogenase complex (NAD+), 2,4-dienoyl-CoA reductase (NADPH) activity, NADH dehydrogenase activity, and NAD binding (Fig. 3D). The upregulated genes included *Card14*, *Sfrp4*, *Lox*, *Col3a1,* and *Adam8* (Table 2). These genes participate in processes such as collagen receptor activity, the Fc-epsilon receptor I complex, and positive regulation of the Toll-like receptor 7 signaling pathway (Fig. 3E). KEGG analysis showed that salivary secretion function was downregulated compared with that in normal samples (Fig. 4A). Inflammation-associated indexes, such as leukocyte transendothelial migration, cytokine-cytokine receptor interaction pathway, and NF-κB, Th1, Th2, and Th17 cell differentiation, were upregulated (Fig. 4B). There were some overlapping genes in these pathways, such as *Cxcl12*, *Nfkb1*, and *Vcam1* (See Supplementary Table 2).

The NF-κB pathway and inflammation play a vital role in the pathological process of the ligated submandibular gland

**Fig. 5:**
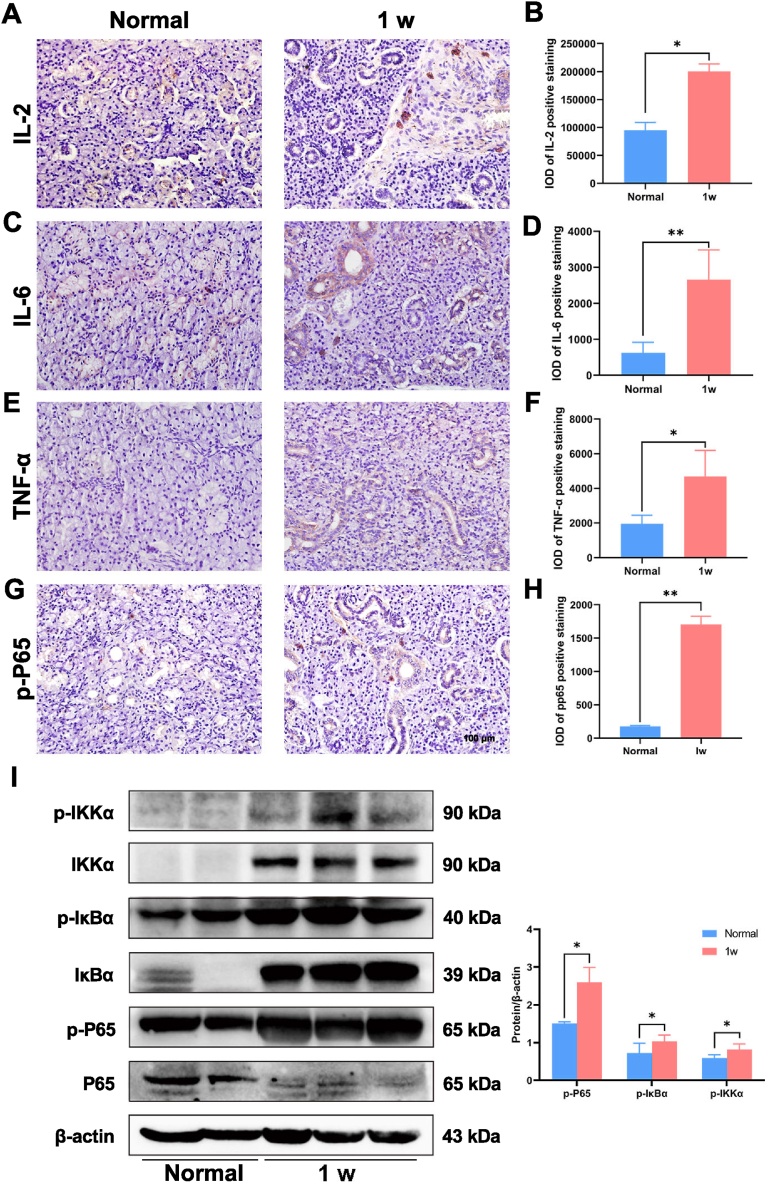
The NF-κB pathway and inflammation in the pathological process of submandibular gland. The inflammatory expression of IL-2 **(A, B)**, IL-6 **(C, D)**, TNF-α **(E, F)**, and p-p65 **(G, H)** in submandibular gland at 1 week and normal group were examined by immunohistochemistry staining and quantified. **I:** p-IκKα, IκKα, p-IκBα, IκBα, p-p65, and p65 were examined by Western blot in 1 week group and normal group. Data were shown as mean ± SEM, N = 6, *p＜0.05, **p＜0.01, T-test.

The inflammatory pathway was examined using samples from the normal group and 1w samples of the submandibular gland after ligation. The levels of IL-2, IL-6, TNF-α, and p-p65 were significantly increased in 1w samples compared with the normal gland using IHC, which confirmed our gene sequencing results (Fig. 5A–H). Members of the NF-κB pathway, such as p-IκKα, IκKα, p-IκBα, IκBα, p-p65, and p65, were significantly upregulated in 1w samples, indicating that inflammatory factors are important in the pathological process of the submandibular gland after duct ligation (Fig. 5I and J).

## 4 Discussion

Ligation of the submandibular gland in rats results in a complete acute injury with rapid pathological changes. Clinical patients mostly experience long-term chronic stimulation, eventually leading to gland atrophy and loss of function. Ligation of the submandibular gland cannot completely simulate clinical pathological changes. However, pathological changes in the submandibular gland after ligation are characteristic. Eventually, it leads to atrophy and hypofunction of the submandibular gland. The pathological changes have certain similarities. In the present study, we explored pathological changes in the rat submandibular gland after duct ligation on day 1 and weeks 1, 2, 3, and 4. In addition, the specific genes between the normal and 1-week samples were screened using RNA sequencing. Our results showed a trend of pathological changes and the vital role of the NF-κB pathway.

The number of acinar cells and secretory function of the submandibular gland detected by AQP5 significantly decreased on day 1 after duct ligation. Although the submandibular gland partially recovered from the injury at week 1, it gradually became damaged over time. These results show that the salivary glands were injured immediately and severely within a short time after ligation. However, based on self-repairing capacity, the injury was slightly alleviated after the acute phase [13]. During the chronic phase, the damage to the submandibular gland progressively worsened. These pathological changes indicate that acute injury to the submandibular gland is rapid and serious.

Fibrosis of the salivary gland after ligation is associated with the TGF-β pathway, which drives fibrosis in most forms of chronic kidney disease. Inhibition of the TGF-β pathway can alleviate renal fibrosis by limiting the proliferation of fibroblast-type cells [22]. In addition, the TGF-β/SMAD pathway plays a vital role in the pathogenesis of hepatic fibrosis, and targeting TGF-β/SMAD signaling is a potential treatment for hepatic fibrosis [23].

Fibrosis is a common physiological and pathological condition of the salivary gland that includes sialadenitis, radiation-induced salivary gland dysfunction, and Sjögren's syndrome [[24], [25], [26]]. Targeting TGF-β pathways for the treatment of salivary gland fibrosis has been studied; however, satisfactory clinical results have not yet been achieved. TGF-β and CD31 expression in the rat submandibular gland after duct ligation, which induce fibrosis, gradually increased. Inhibition of TGF-β signaling is difficult because of its pleiotropic expression and broad distribution in the salivary glands [27]. Thus, it is important to study the fibrotic mechanism of the salivary glands after duct ligation. Our results confirm the vital role of the TGF-β pathway in fibrotic changes in the rat submandibular gland.

RNA sequencing results showed that inflammatory factors were significantly upregulated in salivary glands 1 week after duct ligation. Inflammation is an important trigger for tissue damage and fibrosis. After salivary duct ligation, different cell types in the immune system are activated, which recruit multiple inflammatory factors [28]. Our sequencing results showed that Th1, Th2, and Th17 cell differentiation and the NF-κB pathway were upregulated in the week-1 samples after duct ligation. Based on our analysis, ligation may reduce oxidative metabolic capacity, activate the NF-κB pathway, and induce inflammation, leading to salivary gland injury. Significantly increased expression of the inflammatory signaling pathway was the major cause of hyposalivation. A previous study showed that although inflammatory reactions in the ligated gland are eliminated by dexamethasone, the injured salivary function did not improved [29]. This suggests that inflammation is not only the cause of injury but may also be a trigger point for repair. This can be further verified by single-cell sequencing or in large animals. Further submandibular gland ligation-deligation models are also required to analyze genetic changes. Changes in injury and regeneration were analyzed in combination. Thus, further studies are needed to elucidate the mechanism underlying the induction of inflammation in ligated salivary glands.

## 5 Conclusions

The clinical changes induced by salivary gland disease are different from those in the present experimental duct ligation model, to some extent. Our results detailed pathological changes in the submandibular gland after ligation. Fibrotic processes in the submandibular gland are associated with the TGF-β pathway. The NF-κB pathway and inflammation play vital roles in the pathological processes of the ligated submandibular gland. Moreover, these data could preliminarily provide a pathological basis that explains the changes in the rat submandibular gland after ligation.

## Declarations

### Author contribution statement

**Yang Yang**: Conceived and designed the experiments, Performed the experiments, Analyzed and interpreted the data, Wrote the paper.**Yang Zi**: Performed the experiments, Analyzed and interpreted the data, Wrote the paper.**Du Conglin**: Performed the experiments, Analyzed and interpreted the data.**Zhang Chunmei**: Contributed reagents, materials, analysis tools or data, Wrote the paper.**Hu Liang**: Conceived and designed the experiments, Analyzed and interpreted the data, Wrote the paper.**Wang Songlin**: Conceived and designed the experiments, Contributed reagents, materials, analysis tools or data, Wrote the paper.

### Funding statement

Dr Yang Yang was supported by Beijing Stomatological Hospital, 10.13039/501100002799Capital Medical University Young Scientist Program [YSP202008].Dr Liang Hu was supported by Beijing Hospitals Authority Youth Programme [QML20211502], 10.13039/501100017584Beijing Talents Fund [2018000021469G284], 10.13039/501100001809National Natural Science Foundation of China [82001065].Songlin Wang was supported by 10.13039/501100001809National Natural Science Foundation of China [91649124, 82030031], the Chinese Research Unit of Tooth Development and Regeneration, 10.13039/501100000691Academy of Medical Sciences.[2019–12M-5-031], 10.13039/501100009592Beijing Municipal Science and Technology Commission [Z181100001718208], 10.13039/501100003213Beijing Municipal Education Commission [119207020201], Beijing Advanced Innovation Center for Big Data-Based Precision Medicine [PXM2021_014226_000026], Beijing Municipal Government Beijing Scholar program [PXM2020_014226_000005; PXM2021_014226_000020].

### Data availability statement

Data will be made available on request.

### Declaration of interest's statement

The authors declare no competing interests.
