# Supplementary material for: The NF-κB pathway plays a vital role in rat salivary gland atrophy model
Source: Heliyon. 2023 Mar 8;9(3):e14288. doi: 10.1016/j.heliyon.2023.e14288 (PMC10025116; doi:10.1016/j.heliyon.2023.e14288)
Supplement: Multimedia component 1 [file mmc1.docx]

**Supplementary Figure 1** Body weight, salivary flow rate and submandibular gland mass after duct ligation. **(A)** The body weight of the rats after ligation at 0d, 1d, 1w, 2w, 3w, 4w. **(B)** The salivary flow rate (SFR) of the rats of each group. **(C)** The mass of the submandibular gland of the rats of each group. Data were shown as mean ± SEM, N=6, **p*＜0.05, ***p*＜0.01, ****p*＜0.001.

**Supplementary Figure 2** The micrograph in a lower magnification **(**H&E staining) of submandibular after ligation at NT, 1d, 1w, 2w, 3w, 4w.
