# Supplementary figures and images for: The NF-κB pathway plays a vital role in rat salivary gland atrophy model
Source: Heliyon. 2023 Mar 8;9(3):e14288. doi: 10.1016/j.heliyon.2023.e14288 (PMC10025116; doi:10.1016/j.heliyon.2023.e14288)

A

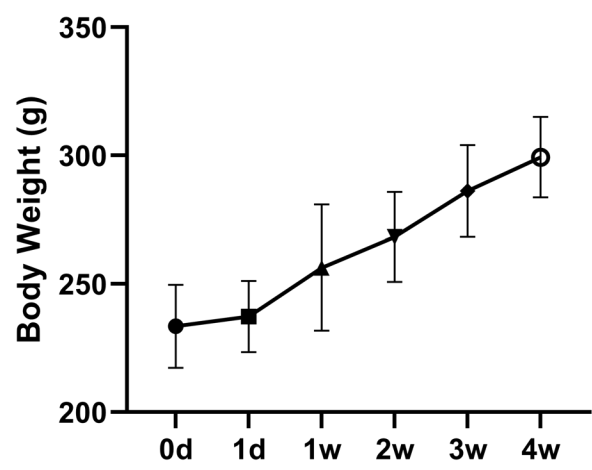

B

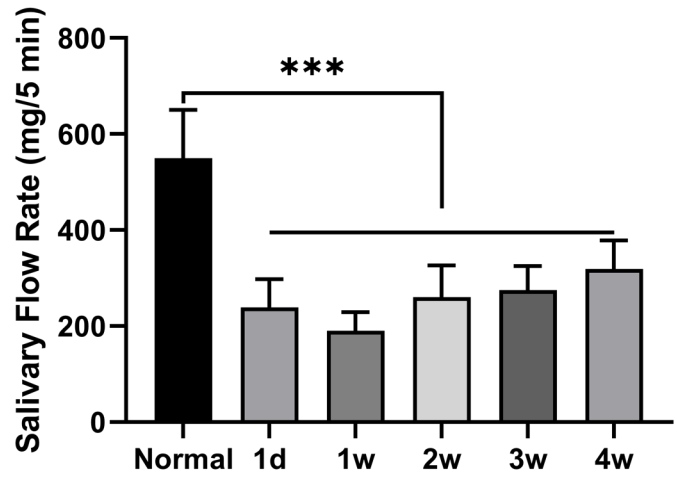

C

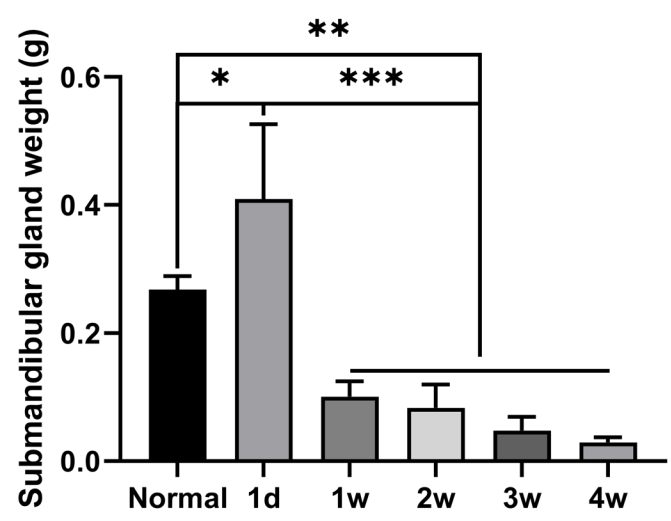

Supplement: Multimedia component 2 [file mmc2.pdf]

# HE Staining

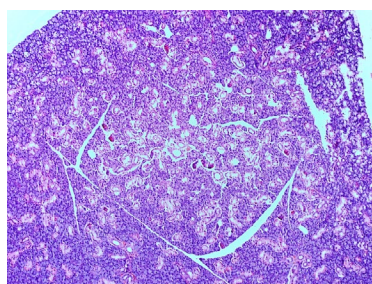

Normal

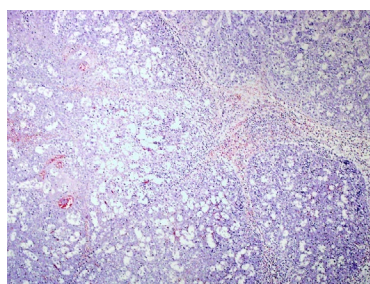

1 d

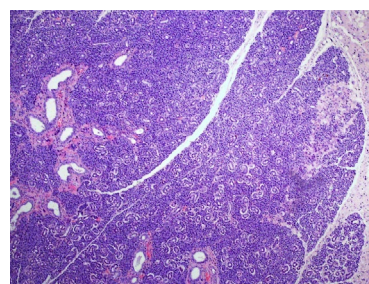

1 w

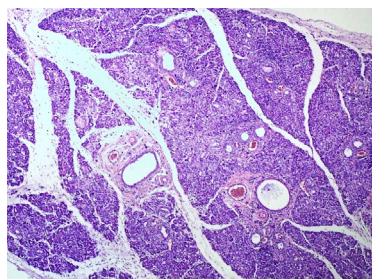

2 w

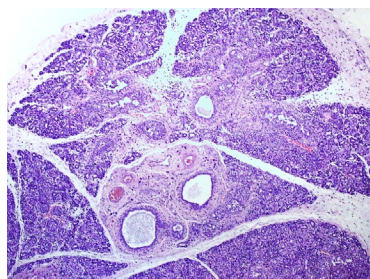

3 w

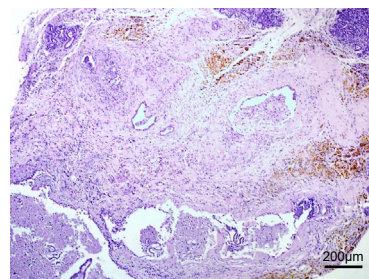

4 w

Supplement: Multimedia component 3 [file mmc3.pdf]

Fig 5l

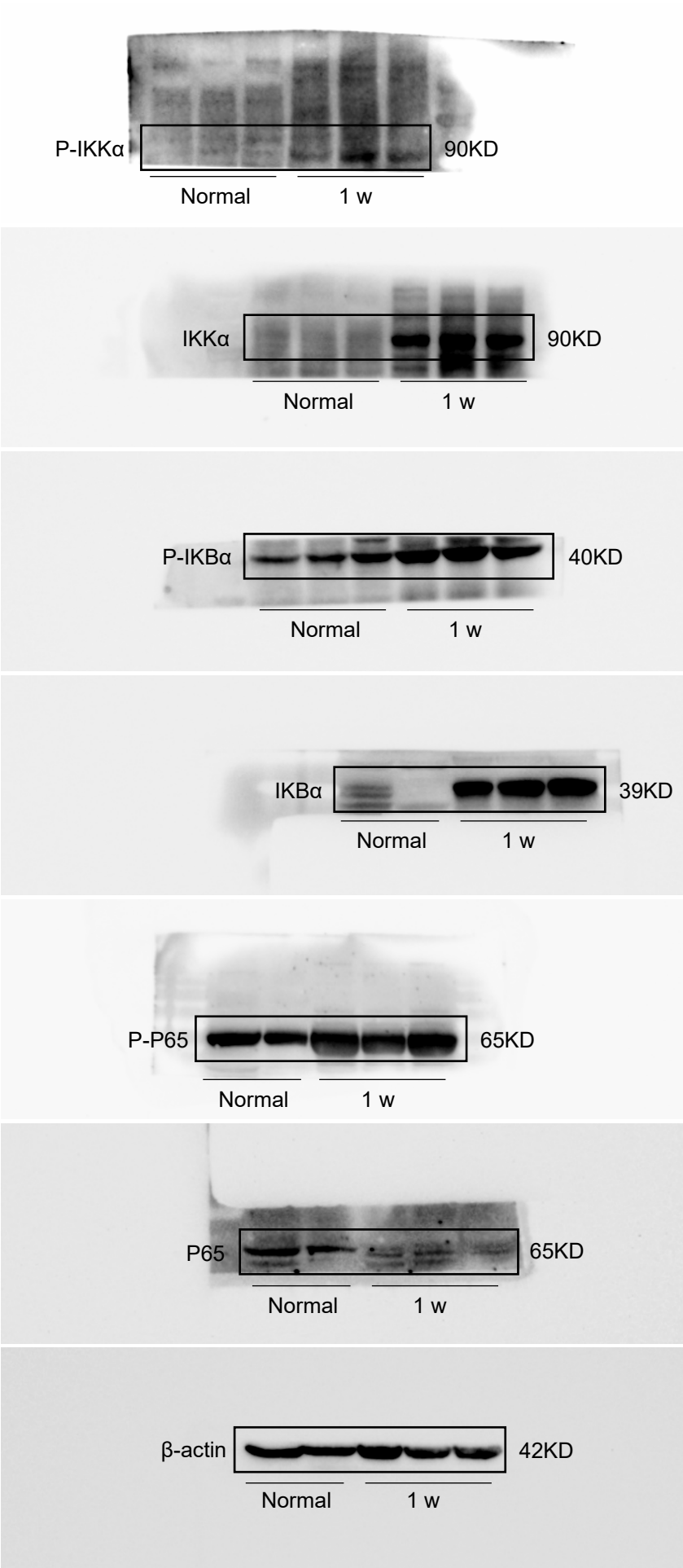

Supplement: Multimedia component 6 [file mmc6.pdf]
