# Supplementary material for: The NF-κB pathway plays a vital role in rat salivary gland atrophy model
Source: Heliyon. 2023 Mar 8;9(3):e14288. doi: 10.1016/j.heliyon.2023.e14288 (PMC10025116; doi:10.1016/j.heliyon.2023.e14288)
Supplement: Multimedia component 4 [file mmc4.docx]

**Supplementary Table 1** PC Values (Group 1w Vs. NT).

| IDs | PC1 | PC2 |
| --- | --- | --- |
| NT1 | -43.4773 | 0.987464 |
| NT2 | -42.5676 | 0.45927 |
| NT3 | -37.1974 | 3.063627 |
| 1w-1 | 33.64462 | -18.2307 |
| 1w-2 | 38.85102 | -7.86375 |
| 1w-3 | 50.74665 | 21.58413 |
