# Supplementary material for: The NF-κB pathway plays a vital role in rat salivary gland atrophy model
Source: Heliyon. 2023 Mar 8;9(3):e14288. doi: 10.1016/j.heliyon.2023.e14288 (PMC10025116; doi:10.1016/j.heliyon.2023.e14288)
Supplement: Multimedia component 5 [file mmc5.docx]

**Supplementary Table 2** Top 30 up-regulated pathway of enrichment (Group 1w Vs. NT).

| Term | ListHits | P  Value | Q  Value | Enrichment_score | Gene |
| --- | --- | --- | --- | --- | --- |
| Cytokine-cytokine receptor interaction | 102 | 5.19E-21 | 1.55E-18 | 2.447790892 | Acvr1; Ccl12; Ccl17; Ccl2; Ccl20; Ccl22; Ccl3; Ccl5; Ccl6; Ccl7; Ccl9; Ccr1; Ccr3; Ccr4; Ccr5; Ccr6; Ccr7; Ccr8; Ccr9; Cd27; Cd40; Clcf1; Crlf2; Csf1; Csf1r; Csf2ra; Csf2rb; Csf3r; Cx3cr1; Cxcl1; Cxcl12; Cxcl13; Cxcl3; Cxcl6; Cxcr2; Cxcr3; Cxcr4; Cxcr6; Edar; Fas; Flt1; Flt3; Flt3lg; Flt4; Hgf; Ifnar1; Ifnar2; Ifngr2; Il10ra; Il10rb; Il12b; Il12rb1; Il13ra1; Il17ra; Il18; Il18r1; Il18rap; Il1a; Il1b; Il1r1; Il1r2; Il21r; Il2ra; Il2rb; Il2rg; Il3ra; Il4r; Il6r; Il7; Il9r; Inhba; Inhbb; Kdr; Lif; Ngfr; Osm; Osmr; Pdgfc; Pdgfra; Pdgfrb; Relt; Tgfb1; Tgfbr1; Tgfbr2; Tnfrsf10b; Tnfrsf11a; Tnfrsf12a; Tnfrsf14; Tnfrsf18; Tnfrsf1a; Tnfrsf1b; Tnfrsf4; Tnfrsf8; Tnfsf10; Tnfsf12; Tnfsf13b; Tnfsf14; Tnfsf18; Tnfsf8; Tslp; Xcl1; Xcr1 |
| Osteoclast differentiation | 61 | 9.20E-18 | 1.37E-15 | 2.915750916 | Acp5; Akt3; Blnk; Btk; Camk4; Creb1; Csf1; Csf1r; Cyba; Fcgr1a; Fcgr2b; Fhl2; Fos; Fosl2; Fyn; Gab2; Ifnar1; Ifnar2; Ifngr2; Il1a; Il1b; Il1r1; Irf9; Itgb3; Junb; Lck; Lcp2; Lilra5; Lilrb1; Lilrb3a; Lilrb3l; Lilrb4; Lilrc2; Map3k14; Mapk11; Ncf1; Ncf2; Ncf4; Nfatc1; Nfkb1; Nfkb2; Nox1; Oscar; Pik3cd; Pirb; Relb; Sirpa; Socs1; Socs3; Spi1; Stat1; Stat2; Syk; Tgfb1; Tgfbr1; Tgfbr2; Tnfrsf11a; Tnfrsf1a; Trem2; Tyk2; Tyrobp |
| Hematopoietic cell lineage | 50 | 3.47E-17 | 3.45E-15 | 3.204121885 | Cd14; Cd19; Cd2; Cd22; Cd33; Cd34; Cd37; Cd38; Cd3d; Cd3e; Cd3g; Cd4; Cd5; Cd55; Cd7; Cd8a; Cd8b; Cr2; Csf1; Csf1r; Csf2ra; Csf3r; Dntt; Fcgr1a; Flt3; Flt3lg; Il1a; Il1b; Il1r1; Il1r2; Il2ra; Il3ra; Il4r; Il6r; Il7; Il9r; Itga2; Itga4; Itga5; Itgam; Itgb3; Mme; RGD1565355; RT1-Ba; RT1-Bb; RT1-DOa; RT1-DOb; RT1-Da; RT1-Db1; Siglech |
| Chemokine signaling pathway | 74 | 3.09E-16 | 2.30E-14 | 2.494399627 | Adcy3; Adcy4; Adcy7; Akt3; Arrb2; Ccl12; Ccl17; Ccl2; Ccl20; Ccl22; Ccl3; Ccl5; Ccl6; Ccl7; Ccl9; Ccr1; Ccr3; Ccr4; Ccr5; Ccr6; Ccr7; Ccr8; Ccr9; Cdc42; Cx3cr1; Cxcl1; Cxcl12; Cxcl13; Cxcl3; Cxcl6; Cxcr2; Cxcr3; Cxcr4; Cxcr6; Dock2; Gnai2; Gnb4; Gng10; Gng2; Gng8; Gngt2; Grk3; Grk4; Grk5; Grk6; Hck; Itk; Jak2; Jak3; Kras; Lyn; Ncf1; Nfkb1; Nras; Pak1; Pik3cd; Plcb2; Prex1; Prkcb; Rac2; Rap1b; Rasgrp2; Rock2; Shc1; Shc2; Shc3; Stat1; Stat2; Tiam1; Vav1; Vav3; Was; Xcl1; Xcr1 |
| Measles | 59 | 9.71E-15 | 5.79E-13 | 2.646604677 | Adar; Akt3; Bbc3; Cblb; Ccnd1; Ccnd2; Ccne1; Ccne2; Cd209e; Cd28; Cd3d; Cd3e; Cd3g; Cdk6; Ddx58; Dok1; Eif2ak2; Fas; Fcgr2b; Fyn; Ifih1; Ifnar1; Ifnar2; Ifngr2; Ikbke; Il12b; Il1a; Il1b; Il2ra; Il2rb; Il2rg; Irak4; Irf7; Irf9; Jak2; Jak3; Msn; Myd88; Nfkb1; Oas1a; Oas1b; Oas2; Oas3; Pik3cd; Prkcq; RGD1564571; Sh2d1a; Slamf1; Stat1; Stat2; Tlr2; Tlr4; Tlr7; Tlr9; Tnfaip3; Tnfrsf10b; Tnfsf10; Tp53; Tyk2 |
| Pathways in cancer | 156 | 1.24E-14 | 6.15E-13 | 1.7596021 | Adcy3; Adcy4; Adcy7; Agtr1a; Akt3; Apaf1; Bak1; Bax; Bbc3; Bcl2l11; Bdkrb1; Bdkrb2; Bid; Birc3; Birc5; Brca2; Calm3; Calml5; Casp3; Cbl; Ccnd1; Ccnd2; Ccne1; Ccne2; Cdc42; Cdk6; Cdkn1a; Cdkn2a; Cdkn2b; Cks2; Col4a1; Col4a2; Crebbp; Csf1r; Csf2ra; Csf2rb; Csf3r; Cxcl12; Cxcr4; Dapk1; Dll1; E2f2; E2f3; Ednra; Ets1; F2r; Fas; Fgf7; Flt3; Flt3lg; Flt4; Fn1; Fos; Fzd1; Fzd2; Gadd45a; Gadd45b; Gli2; Gli3; Gnai2; Gnb4; Gng10; Gng2; Gng8; Gngt2; Gstp1; Hdac1; Hey1; Heyl; Hgf; Hif1a; Hmox1; Ifnar1; Ifnar2; Ifngr2; Igf1; Igf2; Il12b; Il12rb1; Il13ra1; Il2ra; Il2rb; Il2rg; Il3ra; Il4r; Il6r; Il7; Itga2; Itgav; Itgb1; Jag1; Jag2; Jak2; Jak3; Kif7; Kras; Lama3; Lama4; Lamb1; Lamc1; Lamc2; Lef1; Lpar1; Lpar4; Lpar5; Lpar6; Mgst1; Mmp2; Mmp9; Nfkb1; Nfkb2; Notch1; Notch3; Notch4; Nras; Pdgfra; Pdgfrb; Peg12; Pik3cd; Pim1; Plcb2; Pmaip1; Polk; Prkcb; Prkcg; Pten; Ptger2; Ptger4; Rac2; Ralb; Ralgds; Rara; Rasgrp2; Rasgrp3; Rasgrp4; Rb1; Rock2; Runx1t1; Skp2; Spi1; Stat1; Stat2; Stat4; Stat6; Tcf7; Tgfb1; Tgfbr1; Tgfbr2; Tp53; Tpm3; Traf1; Txnrd1; Wnt2; Wnt2b; Wnt5b; Wnt7b |
| Focal adhesion | 77 | 3.33E-14 | 1.42E-12 | 2.279317975 | Actb; Actg1; Akt3; Birc3; Capn2; Cav1; Ccnd1; Ccnd2; Cdc42; Col1a1; Col1a2; Col4a1; Col4a2; Col6a1; Col6a2; Col6a3; Col6a4; Col6a5; Col6a6; Col9a3; Flna; Flnc; Flt1; Flt4; Fn1; Fyn; Hgf; Ibsp; Igf1; Itga11; Itga2; Itga4; Itga5; Itga7; Itga8; Itga9; Itgav; Itgb1; Itgb3; Itgb7; Kdr; Lama3; Lama4; Lamb1; Lamc1; Lamc2; Myl12a; NEWGENE_621351; Pak1; Pak3; Parvb; Parvg; Pdgfc; Pdgfra; Pdgfrb; Pik3cd; Prkcb; Prkcg; Pten; Rac2; Rap1b; Rasgrf1; Rock2; Shc1; Shc2; Shc3; Thbs2; Thbs3; Tln1; Tln2; Tnc; Tnn; Vasp; Vav1; Vav3; Vwf; Zyx |
| Cell adhesion molecules (CAMs) | 69 | 5.09E-14 | 1.89E-12 | 2.380909032 | Cd2; Cd22; Cd226; Cd274; Cd276; Cd28; Cd34; Cd4; Cd40; Cd6; Cd80; Cd8a; Cd8b; Cd99; Cdh2; Cdh5; Cldn1; Cldn11; Cldn2; Cldn5; Cntn1; Ctla4; Esam; Icam1; Icam2; Icos; Icoslg; Itga4; Itga8; Itga9; Itgal; Itgam; Itgav; Itgb1; Itgb2; Itgb7; Lrrc4; Ncam1; Nectin3; Nlgn2; Nlgn3; Nrcam; Pdcd1lg2; Pecam1; Ptprc; Ptprm; RT1-Ba; RT1-Bb; RT1-DOa; RT1-DOb; RT1-Da; RT1-Db1; RT1-M2; RT1-M3-1; RT1-N3; RT1-S3; RT1-T24-3; RT1-T24-4; Sdc1; Sdc3; Sele; Sell; Selp; Selplg; Siglec1; Spn; Tigit; Vcam1; Vcan |
| PI3K-Akt signaling pathway | 111 | 2.02E-13 | 6.67E-12 | 1.915079004 | Akt3; Angpt2; Bcl2l11; Brca1; Ccnd1; Ccnd2; Ccne1; Ccne2; Cd19; Cdk6; Cdkn1a; Col1a1; Col1a2; Col4a1; Col4a2; Col6a1; Col6a2; Col6a3; Col6a4; Col6a5; Col6a6; Col9a3; Creb1; Creb5; Csf1; Csf1r; Csf3r; Ddit4; Efna2; F2r; Fgf7; Flt1; Flt3; Flt3lg; Flt4; Fn1; Gnb4; Gng10; Gng2; Gng8; Gngt2; Hgf; Ibsp; Ifnar1; Ifnar2; Igf1; Igf2; Il2ra; Il2rb; Il2rg; Il3ra; Il4r; Il6r; Il7; Irs1; Itga11; Itga2; Itga4; Itga5; Itga7; Itga8; Itga9; Itgav; Itgb1; Itgb3; Itgb7; Jak2; Jak3; Kdr; Kras; Lama3; Lama4; Lamb1; Lamc1; Lamc2; Lpar1; Lpar4; Lpar5; Lpar6; Mcl1; NEWGENE_621351; Nfkb1; Ngfr; Nos3; Nras; Osm; Osmr; Pdgfc; Pdgfra; Pdgfrb; Phlpp2; Pik3cd; Pik3cg; Pik3r5; Pik3r6; Prkaa1; Pten; RGD1560020_predicted; Sgk3; Syk; Tek; Thbs2; Thbs3; Tlr2; Tlr4; Tnc; Tnn; Tp53; Vwf; Ywhab; Ywhag |
| Th1 and Th2 cell differentiation | 44 | 6.45E-13 | 1.92E-11 | 2.850956451 | Cd247; Cd3d; Cd3e; Cd3g; Cd4; Dll1; Fos; Gata3; Ifngr2; Il12b; Il12rb1; Il2ra; Il2rb; Il2rg; Il4r; Jag1; Jag2; Jak2; Jak3; Lat; Lck; Maf; Maml2; Mapk11; Nfatc1; Nfkb1; Nfkbie; Notch1; Notch3; Prkcq; RT1-Ba; RT1-Bb; RT1-DOa; RT1-DOb; RT1-Da; RT1-Db1; Rbpj; Runx3; Stat1; Stat4; Stat6; Tbx21; Tyk2; Zap70 |
| Complement and coagulation cascades | 41 | 7.86E-13 | 2.08E-11 | 2.951747841 | A2m; Bdkrb1; Bdkrb2; C1qa; C1qb; C1qc; C1r; C1s; C2; C3; C3ar1; C4a; C4bpa; C5ar1; C6; C7; Cd55; Cfd; Cfh; Cfi; Clu; Cr2; F10; F2r; F2rl2; Itgam; Itgax; Itgb2; Kng1; Kng1l1; Masp1; Plat; Plaur; Procr; Pros1; Serpinb2; Serpine1; Serping1; Tfpi; Thbd; Vwf |
| Pertussis | 38 | 8.37E-13 | 2.08E-11 | 3.077737078 | C1qa; C1qb; C1qc; C1r; C1s; C2; C3; C4a; C4bpa; Calm3; Calml5; Casp1; Casp3; Cd14; Cfl1; Cfl2; Cxcl6; Fos; Gnai2; Il12b; Il1a; Il1b; Irak4; Irf1; Irf8; Itga5; Itgam; Itgb1; Itgb2; Mapk11; Myd88; Nfkb1; Nlrp3; Pycard; Serping1; Ticam2; Tirap; Tlr4 |
| Tuberculosis | 68 | 9.61E-13 | 2.20E-11 | 2.278977727 | Akt3; Apaf1; Bax; Bid; C3; Calm3; Calml5; Camp; Card9; Casp3; Cd14; Cd209e; Cd74; Cebpb; Ciita; Clec4e; Clec7a; Coro1a; Creb1; Crebbp; Ctss; Fcer1g; Fcgr1a; Fcgr2b; Ifngr2; Il10ra; Il10rb; Il12b; Il18; Il1a; Il1b; Irak4; Itgam; Itgax; Itgb2; Jak2; Ksr1; Lamp2; Lbp; Lsp1; Mapk11; Mrc1; Mrc2; Myd88; Nfkb1; Nfya; Nod2; Plk3; RGD1564571; RT1-Ba; RT1-Bb; RT1-DOa; RT1-DOb; RT1-Da; RT1-Db1; Rfx5; Sphk1; Stat1; Syk; Tgfb1; Tirap; Tlr1; Tlr2; Tlr4; Tlr6; Tlr9; Tnfrsf1a; Vdr |
| Staphylococcus aureus infection | 30 | 1.42E-12 | 3.03E-11 | 3.498901099 | C1qa; C1qb; C1qc; C1r; C1s; C2; C3; C3ar1; C4a; C5ar1; Cfd; Cfh; Cfi; Fcar; Fcgr1a; Fcgr2b; Icam1; Itgal; Itgam; Itgb2; Masp1; Ptafr; RT1-Ba; RT1-Bb; RT1-DOa; RT1-DOb; RT1-Da; RT1-Db1; Selp; Selplg |
| ECM-receptor interaction | 41 | 2.28E-12 | 4.54E-11 | 2.880621387 | Agrn; Col1a1; Col1a2; Col4a1; Col4a2; Col6a1; Col6a2; Col6a3; Col6a4; Col6a5; Col6a6; Col9a3; Fn1; Gp6; Hspg2; Ibsp; Itga11; Itga2; Itga4; Itga5; Itga7; Itga8; Itga9; Itgav; Itgb1; Itgb3; Itgb7; Lama3; Lama4; Lamb1; Lamc1; Lamc2; NEWGENE_621351; RGD1565355; Sdc1; Sv2a; Thbs2; Thbs3; Tnc; Tnn; Vwf |
| NOD-like receptor signaling pathway | 60 | 2.17E-11 | 4.04E-10 | 2.272013701 | Aim2; Antxr1; Antxr2; Birc3; Camp; Card9; Casp1; Casp12; Casp4; Ccl12; Ccl2; Ccl5; Cxcl1; Cxcl3; Cyba; Cybb; Gbp2; Gbp3; Gbp4; Gbp5; Gsdmd; Ifnar1; Ifnar2; Ikbke; Il18; Il1b; Irak4; Irf7; Irf9; Mapk11; Myd88; Naip5; Naip6; Nampt; Nfkb1; Nlrc4; Nlrp12; Nlrp1a; Nlrp3; Nod2; Oas1a; Oas1b; Oas2; Oas3; Panx1; Plcb2; Pstpip1; Pycard; Ripk3; Rnasel; Rnf31; Stat1; Stat2; Tlr4; Tmem173; Tnfaip3; Trip6; Trpm2; Txn1; Tyk2 |
| HTLV-I infection | 92 | 4.19E-11 | 7.34E-10 | 1.895753246 | Adcy3; Adcy4; Adcy7; Akt3; Anapc4; Bax; Bub1b; Ccnb2; Ccnd1; Ccnd2; Cd3d; Cd3e; Cd3g; Cd40; Cdc20; Cdkn1a; Cdkn2a; Cdkn2b; Creb1; Crebbp; E2f2; E2f3; Egr1; Egr2; Elk4; Ets1; Fos; Fzd1; Fzd2; Icam1; Il1r1; Il1r2; Il2ra; Il2rb; Il2rg; Itgal; Itgb2; Jak3; Kras; Lck; Mad2l1; Map3k14; Map3k3; Mras; Mybl2; Nfatc1; Nfatc4; Nfkb1; Nfkb2; Nras; Nrp1; Pcna; Pdgfra; Pdgfrb; Pik3cd; Pole; Pole2; Pttg1; RGD1560020_predicted; RT1-Ba; RT1-Bb; RT1-DOa; RT1-DOb; RT1-Da; RT1-Db1; RT1-M2; RT1-M3-1; RT1-N3; RT1-S3; RT1-T24-3; RT1-T24-4; Ranbp1; Rb1; Relb; Rras2; Spi1; Tcf3; Tgfb1; Tgfbr1; Tgfbr2; Tln1; Tln2; Tnfrsf1a; Tp53; Tp53inp1; Tspo; Vcam1; Wnt2; Wnt2b; Wnt5b; Wnt7b; Xpo1 |
| Influenza A | 61 | 6.07E-11 | 1.01E-09 | 2.209451004 | Actb; Actg1; Adar; Akt3; Casp1; Ccl12; Ccl2; Ccl5; Ciita; Crebbp; Ddx58; Eif2ak2; Fas; Icam1; Ifih1; Ifnar1; Ifnar2; Ifngr2; Ikbke; Il12b; Il18; Il1a; Il1b; Il33; Irak4; Irf7; Irf9; Jak2; Kpna2; Mapk11; Myd88; Nfkb1; Nlrp3; Oas1a; Oas1b; Oas2; Oas3; Pik3cd; Prkcb; Pycard; RT1-Ba; RT1-Bb; RT1-DOa; RT1-DOb; RT1-Da; RT1-Db1; Rnasel; Rsad2; Socs3; Stat1; Stat2; Tlr3; Tlr4; Tlr7; Tmprss4; Tnfrsf10b; Tnfrsf1a; Tnfsf10; Trim25; Tyk2; Xpo1 |
| Th17 cell differentiation | 45 | 8.14E-11 | 1.28E-09 | 2.523245985 | Cd247; Cd3d; Cd3e; Cd3g; Cd4; Fos; Foxp3; Gata3; Hif1a; Ifngr2; Il12rb1; Il1b; Il1r1; Il21r; Il27ra; Il2ra; Il2rb; Il2rg; Il4r; Il6r; Irf4; Jak2; Jak3; Lat; Lck; Mapk11; Nfatc1; Nfkb1; Nfkbie; Prkcq; RT1-Ba; RT1-Bb; RT1-DOa; RT1-DOb; RT1-Da; RT1-Db1; Rara; Stat1; Stat6; Tbx21; Tgfb1; Tgfbr1; Tgfbr2; Tyk2; Zap70 |
| Hepatitis B | 53 | 1.45E-10 | 2.15E-09 | 2.30648953 | Akt3; Apaf1; Bax; Birc5; Casp12; Casp3; Ccna2; Ccnd1; Ccne1; Ccne2; Cdk6; Cdkn1a; Creb1; Creb5; Crebbp; Ddx58; E2f2; E2f3; Egr2; Egr3; Fas; Fos; Hspg2; Ifih1; Ifnar1; Ikbke; Irf7; Kras; Mmp9; Myd88; Nfatc1; Nfatc4; Nfkb1; Nras; Pcna; Pik3cd; Prkcb; Prkcg; Pten; Rb1; Stat1; Stat2; Stat4; Stat6; Tgfb1; Tgfbr1; Ticam2; Tirap; Tlr2; Tlr3; Tlr4; Tp53; Ywhab |
| AGE-RAGE signaling pathway in diabetic complications | 43 | 2.59E-10 | 3.54E-09 | 2.507545788 | Agtr1a; Akt3; Bax; Casp3; Ccl12; Ccl2; Ccnd1; Cdc42; Col1a1; Col1a2; Col3a1; Col4a1; Col4a2; Cybb; Egr1; Fn1; Icam1; Il1a; Il1b; Jak2; Kras; Mapk11; Mmp2; NEWGENE_621351; Nfatc1; Nfkb1; Nos3; Nox1; Nox4; Nras; Pik3cd; Pim1; Plcb2; Plce1; Prkcb; Sele; Serpine1; Stat1; Tgfb1; Tgfbr1; Tgfbr2; Thbd; Vcam1 |
| Leishmaniasis | 33 | 2.62E-10 | 3.54E-09 | 2.872232245 | C3; Cyba; Cybb; Fcgr1a; Fos; Ifngr2; Il12b; Il1a; Il1b; Irak4; Itga4; Itgam; Itgb1; Itgb2; Jak2; Mapk11; Marcksl1; Myd88; Ncf1; Ncf2; Ncf4; Nfkb1; Prkcb; RT1-Ba; RT1-Bb; RT1-DOa; RT1-DOb; RT1-Da; RT1-Db1; Stat1; Tgfb1; Tlr2; Tlr4 |
| Inflammatiory bowel disease (IBD) | 30 | 1.55E-09 | 2.00E-08 | 2.86795172 | Foxp3; Ifngr2; Il12b; Il12rb1; Il18; Il18r1; Il18rap; Il1a; Il1b; Il21r; Il2rg; Il4r; Maf; Nfatc1; Nfkb1; Nod2; RT1-Ba; RT1-Bb; RT1-DOa; RT1-DOb; RT1-Da; RT1-Db1; Stat1; Stat4; Stat6; Tbx21; Tgfb1; Tlr2; Tlr4; Tlr5 |
| Natural killer cell mediated cytotoxicity | 52 | 1.70E-09 | 2.01E-08 | 2.197377502 | Bid; Casp3; Cd244; Cd247; Cd48; Fas; Fcer1g; Fyn; Hcst; Icam1; Icam2; Ifnar1; Ifnar2; Ifngr2; Itgal; Itgb2; Klrc1; Klrc3; Klrd1; Klrk1; Kras; Lat; Lck; Lcp2; Nfatc1; Nras; Pak1; Pik3cd; Prf1; Prkcb; Prkcg; RT1-M2; RT1-M3-1; RT1-N3; RT1-S3; RT1-T24-3; RT1-T24-4; Rac2; Raet1e; Sh2d1a; Sh2d1b; Sh3bp2; Shc1; Shc2; Shc3; Syk; Tnfrsf10b; Tnfsf10; Tyrobp; Vav1; Vav3; Zap70 |
| Malaria | 28 | 1.71E-09 | 2.01E-08 | 2.968764569 | Ackr1; Ccl12; Ccl2; Cd40; Gypc; Hgf; Icam1; Il18; Il1b; Itgal; Itgb2; Klrb1b; Klrb1c; Klrk1; Lrp1; Myd88; Pecam1; RGD1565355; Sdc1; Sele; Selp; Tgfb1; Thbs2; Thbs3; Tlr2; Tlr4; Tlr9; Vcam1 |
| NF-kappa B signaling pathway | 39 | 1.76E-09 | 2.01E-08 | 2.499215071 | Bcl2a1; Birc3; Blnk; Btk; Card11; Card14; Cd14; Cd40; Cxcl12; Ddx58; Gadd45b; Icam1; Il1b; Il1r1; Irak4; Lat; Lbp; Lck; Lyn; Map3k14; Myd88; Nfkb1; Nfkb2; Prkcb; Prkcq; Relb; Syk; Ticam2; Tirap; Tlr4; Tnfaip3; Tnfrsf11a; Tnfrsf1a; Tnfsf13b; Tnfsf14; Traf1; Trim25; Vcam1; Zap70 |
| Human papillomavirus infection | 101 | 1.94E-09 | 2.15E-08 | 1.727219018 | Akt3; Bak1; Bax; Casp3; Ccna2; Ccnd1; Ccnd2; Ccne1; Ccne2; Cdc42; Cdk6; Cdkn1a; Col1a1; Col1a2; Col4a1; Col4a2; Col6a1; Col6a2; Col6a3; Col6a4; Col6a5; Col6a6; Col9a3; Creb1; Creb5; Crebbp; Eif2ak2; Fas; Fn1; Fzd1; Fzd2; Hdac1; Hdac7; Hdac9; Hey1; Heyl; Ibsp; Ifnar1; Ifnar2; Ikbke; Irf1; Irf9; Isg15; Itga11; Itga2; Itga4; Itga5; Itga7; Itga8; Itga9; Itgav; Itgb1; Itgb3; Itgb7; Jag1; Kras; Lama3; Lama4; Lamb1; Lamc1; Lamc2; Lfng; Maml2; Mfng; NEWGENE_621351; Nfkb1; Notch1; Notch3; Notch4; Nras; Oasl; Oasl2; Pdgfrb; Pik3cd; Pten; Ptger4; RT1-M2; RT1-M3-1; RT1-N3; RT1-S3; RT1-T24-3; RT1-T24-4; Rb1; Rbl1; Rbpj; Stat1; Stat2; Tcf7; Thbs2; Thbs3; Tlr3; Tnc; Tnfrsf1a; Tnn; Tp53; Tyk2; Vwf; Wnt2; Wnt2b; Wnt5b; Wnt7b |
| Leukocyte transendothelial migration | 45 | 2.29E-09 | 2.43E-08 | 2.32227949 | Actb; Actg1; Cd99; Cdc42; Cdh5; Cldn1; Cldn11; Cldn2; Cldn5; Cxcl12; Cxcr4; Cyba; Cybb; Esam; Gnai2; Icam1; Itga4; Itgal; Itgam; Itgb1; Itgb2; Itk; Mapk11; Mmp2; Mmp9; Msn; Myl12a; Ncf1; Ncf2; Ncf4; Pecam1; Pik3cd; Prkcb; Prkcg; Rac2; Rap1b; Rhoh; Rock2; Sipa1; Thy1; Txk; Vasp; Vav1; Vav3; Vcam1 |
| Rap1 signaling pathway | 69 | 2.96E-09 | 3.04E-08 | 1.943833944 | Actb; Actg1; Adcy3; Adcy4; Adcy7; Adora2a; Adora2b; Akt3; Angpt2; Apbb1ip; Arap3; Calm3; Calml5; Cdc42; Csf1; Csf1r; Efna2; F2r; Fgf7; Flt1; Flt4; Fyb; Gnai2; Hgf; Igf1; Itgal; Itgam; Itgb1; Itgb2; Itgb3; Kdr; Kras; Lat; Lcp2; Lpar1; Lpar4; Lpar5; Mapk11; Mras; Ngfr; Nras; P2ry1; Pdgfc; Pdgfra; Pdgfrb; Pfn1; Pik3cd; Plcb2; Plce1; Prkcb; Prkcg; Prkd2; Prkd3; Rac2; Ralb; Ralgds; Rap1b; Rapgef2; Rapgef5; Rasgrp2; Rasgrp3; Rgs14; Sipa1; Skap1; Tek; Tiam1; Tln1; Tln2; Vasp |
| C-type lectin receptor signaling pathway | 44 | 3.99E-09 | 3.96E-08 | 2.311586312 | Akt3; Bcl3; Calm3; Calml5; Card9; Casp1; Cblb; Ccl17; Ccl22; Cd209e; Clec4b2; Clec4d; Clec4e; Clec7a; Egr2; Egr3; Fcer1g; Ikbke; Il12b; Il1b; Irf1; Irf9; Kras; Ksr1; Lsp1; Map3k14; Mapk11; Mras; Nfatc1; Nfatc4; Nfkb1; Nfkb2; Nlrp3; Nras; Pak1; Pik3cd; Plk3; Pycard; RGD1564571; Relb; Rras2; Stat1; Stat2; Syk |
